# Individual life histories: neither slow nor fast, just diverse

**DOI:** 10.1098/rspb.2023.0511

**Published:** 2023-07-12

**Authors:** Joanie Van de Walle, Rémi Fay, Jean-Michel Gaillard, Fanie Pelletier, Sandra Hamel, Marlène Gamelon, Christophe Barbraud, F. Guillaume Blanchet, Daniel T. Blumstein, Anne Charmantier, Karine Delord, Benjamin Larue, Julien Martin, James A. Mills, Emmanuel Milot, Francine M. Mayer, Jay Rotella, Bernt-Erik Saether, Céline Teplitsky, Martijn van de Pol, Dirk H. Van Vuren, Marcel E. Visser, Caitlin P. Wells, John Yarrall, Stéphanie Jenouvrier

**Affiliations:** ^1^ Department of Biology, Woods Hole Oceanographic Institution, Woods Hole, MA, USA; ^2^ Centre for Biodiversity Dynamics, Department of Biology, Norwegian University of Science and Technology, Trondheim, Norway; ^3^ Laboratoire de Biométrie et Biologie Évolutive, CNRS-UMR5558, Université de Lyon, Université Lyon 1, Villeurbanne, France; ^4^ Département de Biologie, Université de Sherbrooke, Sherbrooke, Quebec, Canada; ^5^ Département de Mathématiques, Université de Sherbrooke, Sherbrooke, Quebec, Canada; ^6^ Département des Sciences de la Santé Communautaire, Université de Sherbrooke, Sherbrooke, Quebec, Canada; ^7^ Département de Biologie, Université Laval, Quebec, Canada; ^8^ Centre d’Études Biologiques de Chizé, CNRS-UMR7372, Université La Rochelle, Villiers en Bois, France; ^9^ Department of Ecology and Evolutionary Biology, University of California, Los Angeles, CA, USA; ^10^ The Rocky Mountain Biological Laboratory, Crested Butte, CO, USA; ^11^ Centre d’Écologie Fonctionnelle et Évolutive, CNRS, EPHE, IRD, Université de Montpellier, Montpellier, France; ^12^ Department of Biology, University of Ottawa, Ottawa, Ontario, Canada; ^13^ 10527A Skyline Drive, Corning, NY, USA; ^14^ 3 Miromiro Drive, Kaikoura, New Zealand; ^15^ Département de Chimie, Biochimie et Physique and Forensics Research Group, Université du Québec à Trois-Rivières, Trois-Rivières, Quebec, Canada; ^16^ Département des Sciences Biologiques, Université du Québec à Montréal, Montréal, Quebec, Canada; ^17^ Department of Ecology, Montana State University, Bozeman, MT, USA; ^18^ College of Science and Engineering, James Cook University, Townsville, Australia; ^19^ Department of Wildlife, Fish, and Conservation Biology, University of California, Davis, CA, USA; ^20^ Department of Animal Ecology, Netherlands Institute of Ecology (NIOO-KNAW), Wageningen, The Netherlands; ^21^ Department of Fish, Wildlife and Conservation Biology, Colorado State University, Fort Collins, CO, USA; ^22^ 14 Ashgrove Court, Lincoln, New Zealand

**Keywords:** demography, individual heterogeneity, intra-specific variation, pace-of-life syndrome, slow–fast continuum, trade-off

## Abstract

The slow–fast continuum is a commonly used framework to describe variation in life-history strategies across species. Individual life histories have also been assumed to follow a similar pattern, especially in the pace-of-life syndrome literature. However, whether a slow–fast continuum commonly explains life-history variation among individuals within a population remains unclear. Here, we formally tested for the presence of a slow–fast continuum of life histories both within populations and across species using detailed long-term individual-based demographic data for 17 bird and mammal species with markedly different life histories. We estimated adult lifespan, age at first reproduction, annual breeding frequency, and annual fecundity, and identified the main axes of life-history variation using principal component analyses. Across species, we retrieved the slow–fast continuum as the main axis of life-history variation. However, within populations, the patterns of individual life-history variation did not align with a slow–fast continuum in any species. Thus, a continuum ranking individuals from slow to fast living is unlikely to shape individual differences in life histories within populations. Rather, individual life-history variation is likely idiosyncratic across species, potentially because of processes such as stochasticity, density dependence, and individual differences in resource acquisition that affect species differently and generate non-generalizable patterns across species.

## 1. Introduction

Species display an astonishing diversity of life-history traits [1], which are shaped by species differences in their body size or mass, Bauplan and lifestyle [2]. Organisms face time and energy allocation trade-offs because time and resources are limited in nature. Across species, the trade-off between survival and reproduction determines the pace of the life cycle [3], the so-called slow–fast continuum [4]. Along this slow–fast continuum, species rank from a combination of late reproduction, low reproductive rates, and long lifespans at the *slow* end to a combination of opposite characteristics at the *fast* end [5]. The slow–fast continuum has been consistently identified as the driving axis of life-history variation across species in most taxa studied so far, including birds, mammals, insects and plants [1,6–10].

Within a given species, individuals also show a high diversity of life-history traits [11–14]. Two decades ago, the idea that a slow–fast continuum of life-histories could also exist among populations within a given species emerged from the observation that tropical songbird populations had slower life histories than temperate ones [15,16]. Different populations experience different environmental conditions, which can affect the expression of life-history traits. A demonstration of this comes from Reznick *et al*.'s [17] experimental study showing a shift in population-level life histories after the introduction of predators in nature. More recently, yellow-bellied toad [18] and common lizard [19] populations affected by increased anthropization and climate warming, respectively, have been observed accelerating their life cycles through compensatory investment in reproduction. At the population level, identifying patterns of life-history variation has generated much research, but what structures such variation remains unclear. A common finding is that life-history trade-offs are not always detected [20]. Individual differences in resource acquisition can generate positive correlations between life-history traits, hence masking life-history trade-offs. Whether the slow–fast continuum, defined by a series of trade-offs between life-history traits in time units [5], structures individual variation as it does across species is therefore even less clear. The existence of a slow–fast continuum of life histories among individuals has nevertheless been repeatedly assumed in behavioural ecology in regard to the concept of pace-of-life syndrome (POLS) [21,22]. Specifically, the POLS hypothesis posits that a series of behavioural and physiological axes of variation correlates with the individual slow–fast continuum [21]. A formal test of whether the slow–fast continuum drives individual life-history variation within populations is thus needed to move forward in our understanding of how individual heterogeneity in life history is structured within populations.

**Figure 1.**
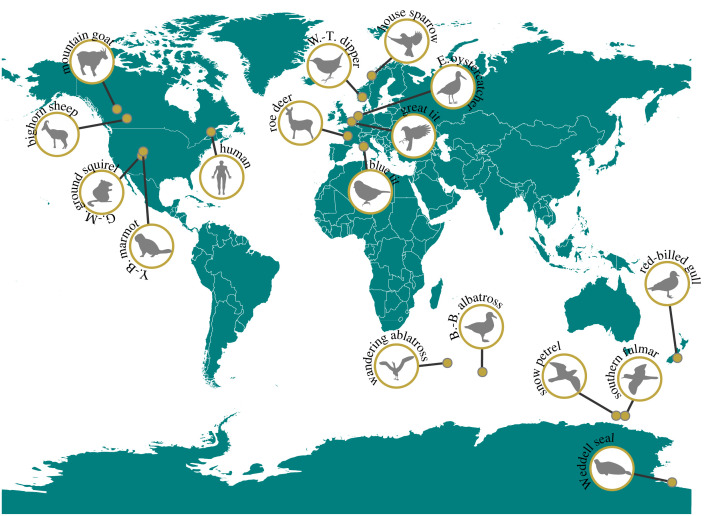
Geographical location of the study populations for the 17 species included in the analyses. Our study includes 10 bird species: white-throated dipper (*Cinclus cinclus*), house sparrow (*Passer domesticus*), great tit (*Parus major*), blue tit (*Cyanistes caeruleus*), Eurasian oystercatcher (*Haematopus ostralegus*), wandering albatross (*Diomedea exulans*), black-browed albatross (*Thalassarche melanophris*), snow petrel (*Pagodroma nivea*), southern fulmar (*Fulmarus glacialoides*) and red-billed gull (*Larus novaehollandiae*) and seven mammal species: bighorn sheep (*Ovis canadensis*), mountain goat (*Oreamnos americanus*), golden-mantled ground squirrel (*Callospermophilus lateralis*), yellow-bellied marmot (*Marmota flaviventer*), human (*Homo sapiens*), and Weddell seal (*Leptonychotes weddellii*).

Here, we empirically assessed whether a slow–fast continuum structures individual life-history variation. Our approach is based on the assessment of a slow–fast continuum both across species and among individuals within populations, using the same set of species and focal life-history traits. We relied on 17 of the world's most detailed, long-term and individual-based population monitoring of bird and mammal species (figure 1) with contrasting life-history strategies (electronic supplementary material table S1). First, using principal component analyses (PCA [23]) and correcting for phylogenetic relatedness, we identified the structuring axes of variation among four life-history traits calculated across individuals for each species. We used age at first reproduction, adult lifespan, breeding frequency and fecundity, which are life-history traits commonly used to describe the slow–fast continuum of life histories in vertebrates [1,5,9,24,25]. We deemed a slow–fast continuum as present if the first component of the PCA was selected and if both traits with a time duration dimension (i.e. age at first reproduction and adult lifespan) covaried negatively with traits with a time frequency dimension (i.e. breeding frequency and fecundity). To determine whether the first component (PC1) of the PCA was selected, we followed the broken-stick model [26], which sets the threshold above which a component explains more variation than what would be expected by chance alone. Then, we tested whether life-history variation across species and among individuals within populations aligned [27] by comparing variation in life-history traits at both levels of biological organization for all species simultaneously. Finally, we used the same procedure for each species separately to assess whether the slow–fast continuum consistently structured life-history variation within populations.

## 2. Methods

### (a) Species and study populations

We analysed individual life-history data from 10 bird and seven mammal species. The 17 species represent a large diversity of life-history strategies and large sample sizes of individuals with complete records of life histories were available for each species. See electronic supplementary materials table S1 for the list of species and figure 1 for the geographical location of the study populations.

### (b) Life-history traits

To compute life-history traits, we conducted a strict filtering of the data. See electronic supplementary materials, S1–S2 for more details on data treatment and permits to carry out research on each species. The slow–fast continuum is a timescale based on the concept of biological time (*sensu* [28]) and, as pointed out by Gaillard *et al*. [5], it should only include traits that either have a dimension of time duration (e.g. years) or time frequency (e.g. number/year). When evaluating relationships among life-history traits and assessing the slow–fast continuum of life-history strategies, respecting dimensionality is key to obtain isometric relationships among traits [5]. Further, because our goal was to assess whether the slow–fast continuum structuring life-history variation among species can be transposed to life-history variation among individuals within a population, we used four life-history traits that had a similar meaning and that could be calculated similarly across levels of biological organization, as outlined in Araya-Ajoy *et al*. [29]. For each individual of each species, we thus calculated (i) age at first reproduction (years), (ii) adult lifespan (years), (iii) fecundity (i.e. number of independent offspring produced per year), and (iv) breeding frequency (number of breeding events per year). We also computed individual generation time. Generation time has often been used to characterize the slow–fast continuum (e.g. [1]). However, generation time implies in its construction other life-history traits [30]. To avoid generating artificial correlations in our analyses, we did not use generation time in the PCA, but used it as a covariate in a subsequent, independent, analysis (see section *Analyses*). More details on trait calculation can be found in electronic supplementary materials, S1. See electronic supplementary materials table S1 for means and ranges of life-history traits and generation times.

### (c) Analyses

#### (i) Retrieving the slow–fast continuum of life histories across species

PCA [23] is the standard tool to identify the structuring factors driving life-history variation across species and to detect the slow–fast continuum [1,4–6,9,10,25,31,32]. Thus, as a first step, to assess whether our selection of life-history traits could allow for the detection of the slow–fast continuum, we conducted a PCA accounting for the non-independence among species due to phylogenetic relatedness (*p*PCA) [33] using the ‘phyl.pca' function in the ‘phytools' [33] R package. See electronic supplementary materials, S4 for phylogenetic trees construction. For each of the 100 trees generated, we conducted one *p*PCA and summarized results across trees. We also conducted one *p*PCA using an average tree, which we computed using the quadratic path difference criterion in the ‘averageTree' function from the ‘phytools' R package. We calculated the proportion of variation explained by each principal component. For each life-history trait, we calculated its relative importance for each principal component by dividing its absolute loading on each principal component by the sum of its absolute loadings across principal components. Loadings were extracted from the summary output of the ‘phyl.pca' from the ‘phytools' R package. Because the slow–fast continuum should represent the main axis structuring individual variation in life-history traits, we present results for the first principal component, PC1, in the main text while results from all other principal components are available in electronic supplementary material, table S6.

Life-history traits at the species level were calculated by taking the mean of individual life-history traits for each species. Individual traits were not all normally distributed for all species, and we checked the robustness of our results to the deviation from normality by conducting a PCA among species using median values and the first and third quartiles of the data distribution in electronic supplementary material, S6. All mean life-history variables were log-transformed to correct for allometric relationships between variables across species and then standardized (mean = 0, standard deviation = 1) prior to analyses. We used the broken-stick model [26] to determine the number of structuring axes in the PCAs and whether the first principal component was retained. See electronic supplementary materials, S3 for more information on the broken-stick model. According to this model, the first principal component was retained if it explained more than 52% of variation. In the context of our study, we deemed a slow–fast continuum as present if four conditions were met: (i) the first component of the PCA was selected according to the broken-stick model, (ii) traits with a time duration dimension (i.e. age at first reproduction and adult lifespan) covaried positively, (iii) traits with a time frequency dimension (i.e. breeding frequency and fecundity) covaried positively, and (iv) traits with a time dimension and traits with a time frequency dimension covaried negatively.

Because generation time is a reliable measure of a species' position along the slow–fast continuum [34], we complemented this analysis by testing whether, and to which extent, species' positions along the main axis of variation in the PCA was correlated with their (log-transformed) generation times using a linear regression.

#### (ii) Detecting the slow–fast continuum among individuals within populations

In a second step, we included individuals' life-history traits onto the *p*PCA performed at the interspecific level to compare patterns of life-history variation at both levels simultaneously. Individuals' data consisted of repeated measures of the same species and therefore could not be directly analysed using a *p*PCA. Instead, we projected individuals' data directly onto the *p*PCA conducted across species with the average phylogenetic tree. To do so, individuals' data for each of the four life-history traits were log-transformed and standardized (mean = 0, standard deviation = 1). Then, for each species, individuals' data were rotated according to the species eigenvectors from the *p*PCA using the ‘scores' function in the ‘phytools' R package. Individuals' rotated scores were then added to the biplot of the *p*PCA and we computed 95% data ellipses for each species using the ‘dataEllipse' function from the ‘car' [35] R package. For each species, we calculated the variance in rotated individual scores along each principal component and across all principal components. To test whether individual life-histories aligned with the slow–fast continuum across species, we compared for each species the proportion of the total variance in rotated individual scores on *p*PC1 with the proportion of variance explained by the *p*PC1 at the interspecific level. A lower proportion would indicate a failure to align with the slow–fast continuum across species.

#### (iii) Species-specific axes of variation in individual life histories

We conducted a separate PCA on individual data within each population and followed the same methodology as described in the section *Retrieving the slow*–*fast continuum of life histories across species*, but this time considering only individual life-history traits. We performed the species-specific PCAs using the ‘vegan' [36] R package. As for the interspecific level, all individual life-history traits were log-transformed and then scaled (mean = 0, standard deviation = 1) prior to analysis. Note that some individuals may have bred in their lifetime, but without success. This led to a null fecundity for these individuals because fecundity only considers the production of independent offspring. To avoid generating infinitesimal values resulting from the log-transformation, we increased all individual fecundity rates by one prior to log-transformation and standardization. For consistency purposes, we also increased fecundity rates by one in the among species analysis above. For each species-specific PCA, we extracted the proportion of variation explained by PC1 and tested whether it was related to a species' (log-transformed) generation time using linear models. As for the *p*PCA across species, we then used the broken-stick model (electronic supplementary material, figure S1) to determine the number of structuring axes of variation in each species. We deemed the slow–fast continuum of life-history strategies present if the first component was retained and was characterized by an opposition of time duration-related traits and time frequency-related traits. For each species, we conducted a linear regression to assess whether, and to which extent, individual scores on PC1 were correlated with individual (log-transformed) generation times.

## 3. Results

### (a) Retrieving the slow–fast continuum of life histories across species

**Figure 2.**
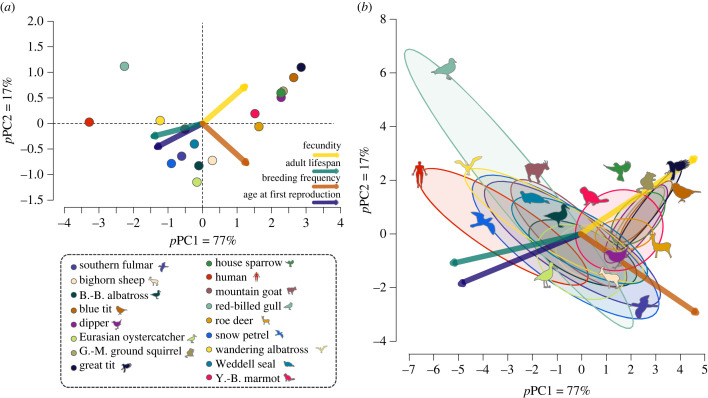
The slow–fast continuum of life histories across species. (*a*) Pattern of covariation among life-history traits across 17 bird and mammal species revealed by a phylogenetically informed PCA (*p*PCA) using species-specific trait values. The *p*PCA was calculated using an average phylogenetic tree for birds and mammals over 100 trees. (*b*) Projection of individual scores (i.e. rotated individual data according to species' means) on the *p*PCA performed on species' mean life-history traits. Different colours for points and 95% data ellipses correspond to different species. Length of eigenvectors was increased by a factor of (*a*) 2 and (*b*) 5.5 for ease of visualization.

According to the broken-stick model (electronic supplementary material, figure S1), the first principal component of the PCA correcting for phylogenetic relatedness among species (*p*PC1) was retained as the main structuring axis of variation in life histories across species. The *p*PC1 accounted for 76–84% (range across 100 phylogenetic trees; table 1) of the overall variation. Using an average phylogenetic tree, *p*PC1 accounted for 77% of variation. No other principal component was retained as a secondary structuring axis (electronic supplementary material, table S6). As expected, *p*PC1 fully aligned with the slow–fast continuum by opposing traits expressed in time units to traits expressed in time frequencies (figure 2*a*). The contributions of all traits to *p*PC1 were high and close to equi-correlation (table 1; electronic supplementary material, table S6), which indicates that all traits were equally important in defining the slow–fast continuum. Not surprisingly, a species' position on *p*PC1 (i.e. on the slow–fast continuum) was closely linked with its generation time (*R*^2^ = 0.78; electronic supplementary material, table S8), which supports the hypothesis that generation time reliably predicts species' position along the slow–fast continuum [34]. When using a simple PCA without accounting for phylogenetic relatedness, the same pattern held among species, with even stronger support for the slow–fast continuum (electronic supplementary material, tables S3–S5 and figure S2).

**Table 1.** Principal component analyses (PCA) across species and among individuals within populations. The proportion of variation explained, the relative importance of each life-history trait and their loadings on PC1 are shown. The relative importance for PC1 (or *p*PC1) for each trait was calculated as the absolute value of the loading of PC1 (or *p*PC1) divided by the total loading across principal components. Because of the random direction of axis rotation in a PCA and for ease of comparison between species, the orientation (sign) of the loadings of life-history traits were reversed to keep ‘adult lifespan' on the negative side of PC1 (or *p*PC1), when necessary. In golden-mantled ground squirrel and blue tit, breeding frequency did not vary among individuals. Similarly, in the great tit, both breeding frequency and age at first reproduction showed no individual variation. For those three species, their respective PCAs were reduced to either two or three dimensions.

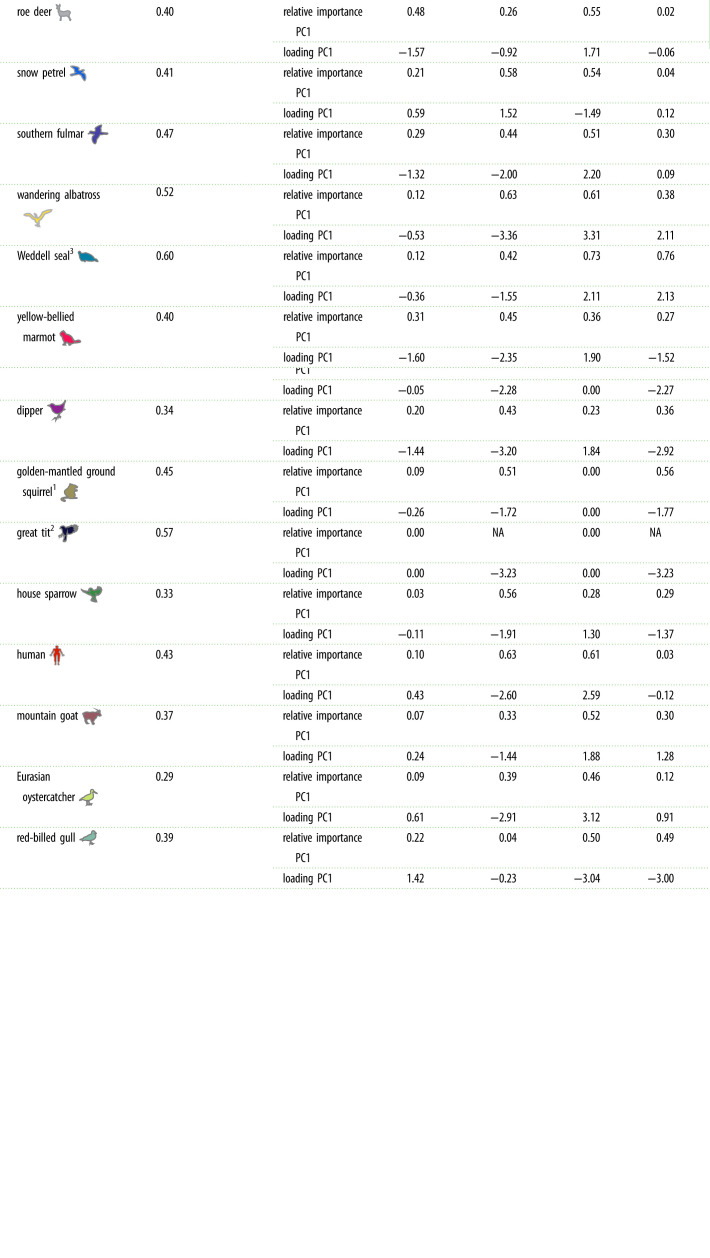

^1^Breeding frequency did not vary across individuals in golden-mantled ground squirrel and blue tit, leading to only three potential components instead of four.

^2^Breeding frequency and age at first reproduction did not vary across individuals in great tit, leading to only two potential components instead of four.

^3^Breeding frequency and fecundity are confounded in the Weddell seal, which artificially inflates the proportion of variation accounted for by PC1.

### (b) Detecting a slow–fast continuum of life histories among individuals within populations

To test the expectation that life-history variation across species and among individuals within populations should align, we added individual data to the *p*PCA across species using an average phylogenetic tree. We found that individual life-history variation within species did not align with the slow–fast continuum; the 95% data ellipses containing individual data for each species was not distributed along the slow–fast continuum across species, i.e. *p*PC1 (figure 2*b*). Moreover, for all species the percentage of the total variance in individual data on *p*PC1 was well below the 77% found across species (range: 12–62%, electronic supplementary material, table S9). This suggests that overall life-history variation among individuals does not align with the slow–fast continuum across species, and that a different and independent pattern might explain life-history traits covariation between individuals within populations.

Individual life-history variation seemed to be structured differently depending on the species' position on the slow–fast continuum (figure 2*b*). In slow species (i.e. human, wandering albatross, southern fulmar, Weddell seal, snow petrel, mountain goat and black-browed albatross), individuals were more largely spread along PC1, compared to species with an intermediate (i.e. yellow-bellied marmot, bighorn sheep, Eurasian oystercatcher and roe deer) or fast (i.e. great tit, blue tit, white-throated dipper, golden-mantled ground squirrel and house sparrow) life cycle speed. Because adult lifespan characterizes the most PC1 (table 1), this suggests that survivorship patterns strongly structure individual life-history variation in slow species. In fast species, individual life histories seemed more spread along other PCs, such as PC2 (figure 2*b*; electronic supplementary material, table S9), which are more characterized by reproductive traits (electronic supplementary material, table S5). Reproductive traits may play a greater role in shaping individual variation in life history in fast species.

### (c) Species-specific axes of variation in individual life histories

**Figure 3.**
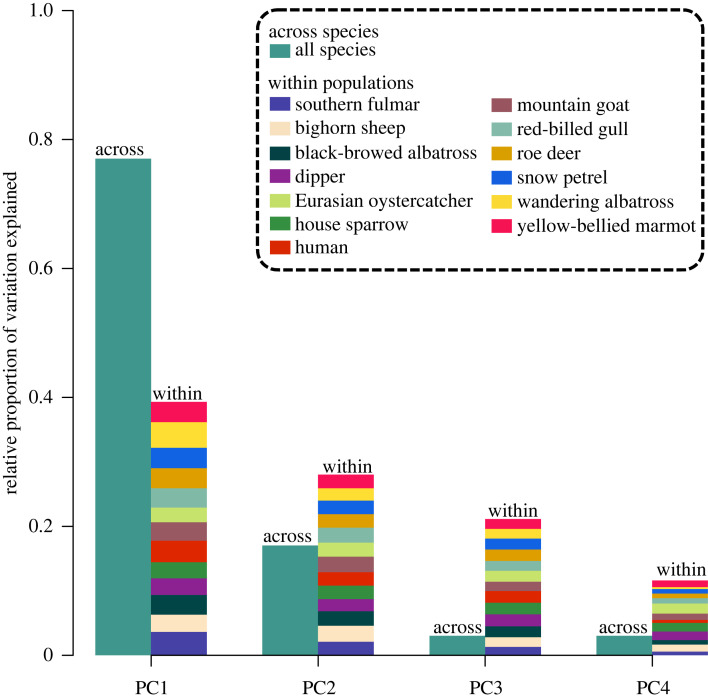
Relative importance of principal components, PC, across species and among individuals within populations. The height of bars labelled ‘across' correspond to the proportion of variation explained by each PC of the *p*PCA across species (table 1). The height of the bars labelled ‘within' was calculated for each PC by summing the proportion of variation explained by this PC for all species and dividing by the total number of species. Each colour-coded rectangle in the ‘within' bars represents the importance of that axis for a given species, standardized by the total importance of that axis across species (i.e. the height of the bar). Within populations, we present only the 13 species for which four distinct components could be extracted. The great tit, the blue tit and the golden-mantled ground squirrel were excluded because their specific PCAs were based on less than four PCs due to the absence of variation in one or two life-history traits. The Weddell seal was also excluded because two life-history traits (breeding frequency and fecundity) were confounded in this species.

Focusing on individual variation in life histories within populations for each species independently, we tested for the presence of a slow–fast continuum in each species using species-specific PCAs (figure 3). In the great tit, there was no variation in age at first reproduction and breeding frequency among individuals. In the blue tit and the golden-mantled ground squirrel, there was also no variation in breeding frequency. For these species, the slow–fast continuum cannot be detected, hence comparison with other species was not possible. Also, breeding frequency and fecundity in the Weddell seal were confounded because of the absence of information on weaning success (electronic supplementary material, S1), which also hindered comparison with other species. Results for these four species are presented for consistency but should be interpreted with caution.

**Figure 4.**
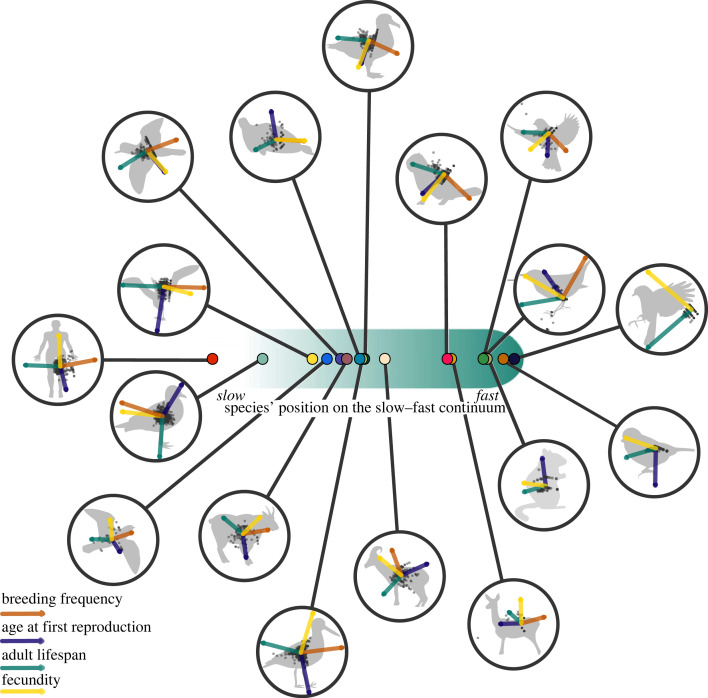
Species' positions on the slow–fast continuum of life histories across species and the species-specific individual patterns of covariation in life-history traits. Individual values (grey dots) and scores of life-history traits on the first and second axes of variation (arrows) extracted from individual-level principal component analyses (PCA) are shown for each species. Species' positions on the slow–fast continuum correspond to the species' scores on the first axis of a phylogenetic PCA at the species level using the species' mean value for each life-history trait. For ease of interpretation and comparison among species, the orientation (sign) of the scores of individual and life-history traits were reversed to show the green arrow (adult lifespan) pointing towards the left-hand side of the plots, when necessary.

For the 13 remaining species, the proportion of variance explained by the main axis, PC1, was consistently much lower than that retrieved across species (figure 3). Indeed, PC1 accounted for between 29% of life-history variation among individuals in the Eurasian oystercatcher and 52% in wandering albatross (table 1). Interestingly, there was a positive relationship between a species' generation time and the proportion of individual variation explained by PC1 (*β* = 0.036, s.e. = 0.016, *p* = 0.048). However, following the broken-stick model (electronic supplementary material, figure S1), the only species that was close to showing a dominantly structuring axis of variation was the wandering albatross (52% of variation explained by PC1). For this species, the pattern of covariation between life-history traits appeared qualitatively in line with the slow–fast continuum (figure 4). However, contrary to the equal contribution of all time and time frequency traits expected from a slow–fast continuum, age at first reproduction had a rather independent role in explaining individual variation in this species. Indeed, age at first reproduction contributed much less to PC1 than other life-history traits (table 1) and mostly contributed to PC2 (electronic supplementary material, table S6).

For all other species, the proportion of life-history variation explained by PC1 was (often far) below the 52% threshold necessary for it to be interpreted as a structuring axis based on the broken-stick model (table 1). Individual variation could thus not be distinguished from a random process and therefore did not seem to be structured by any of the life-history traits considered. When looking at species-specific PCAs (figure 4), we nevertheless noted that across species, adult lifespan and breeding frequency covaried negatively, which might suggest a trade-off between those two life-history traits. Indeed, the covariance between adult lifespan and breeding frequency was negative and strong (less than or equal to −0.5) in six species (electronic supplementary material, table S10). Overall, age at first reproduction was the life-history trait that covaried the least with other life-history traits, suggesting that individual variation in this trait is independent from the other traits we analysed. Although species-specific PC1s could not be interpreted as structuring axes of individual life-history variation, we assessed the relationship between PC1 scores and individual-level generation time in each species to provide a direct comparison with the pattern of life-history variation retrieved across species. The strength of species-specific relationships was much weaker (average *R*^2^ = 0.32, with *R*^2^ values ranging from 0.003 in southern fulmar to 0.69 in yellow-bellied marmot) than that observed across species (0.78 and 0.92 with and without phylogeny included, electronic supplementary material, table S8). Detailed PCAs and correlation plots for each species can be found in electronic supplementary material, figures S3–S6. Our analyses thus provide clear evidence that the slow–fast continuum is not a structuring axis of life-history variation among individuals within a population.

## 4. Discussion

Based on high-quality and long-term demographic data from a substantial number of bird and mammal species with contrasting life-history strategies, we assessed whether the slow–fast continuum of life histories is the main factor structuring individual life-history variation within a given population. Using key demographic traits that shape the speed of a life cycle, namely age at first reproduction, adult lifespan, fecundity and breeding frequency, we successfully retrieved the expected slow–fast continuum across species. The percentage of variance in life-history traits explained by the slow–fast continuum we provided is in line with previous studies (70–80% in vertebrates [5,10,24]). Moreover, as expected from previous work, all time and time frequency traits equally contributed to the slow–fast continuum and species-specific scores on the slow–fast continuum were closely associated with species' generation times. However, individual variation in those same life-history traits within a given population did not align with the slow–fast continuum. The wandering albatross was the only species for which PC1 almost supported expectations from the slow–fast continuum. The apparent breeding frequency–adult lifespan trade-off in the wandering albatross could be explained by their extreme long life and their quasi-biennial breeding tactic. Indeed, most successful breeders, but not failed breeders, skip breeding the following year to replenish body reserves [37], which may exacerbate the trade-off between breeding and survival processes and increase individual variation. Nevertheless, for all species including the wandering albatross, the broken-stick model indicated that no structure could be statistically identified beyond a random variation. As a result, our study showed that individual life histories within a population are not structured by a slow–fast continuum.

Our approach was based on comparing life-history variation across species and among individuals within populations using the same set of species, life-history traits and methodological tools. The approach we used has been previously suggested [27,29], but not yet applied, potentially because of the challenges of monitoring a large number of individuals over their entire lifetime within a population in the wild. In our collaborative effort, we could access the required individual life-history records for 17 bird and mammal species. With this set of species, we first confirmed the existence of the slow–fast continuum as the major structuring axis of life-history variation across species before moving on to the individual level. Based on the same data, our approach would have allowed for the detection of the slow–fast continuum of life histories among individuals within a population, if present in any of the species considered. Many studies have previously acknowledged that the challenges associated with the detection of a slow–fast continuum within populations are great and numerous [24,29]. Below we discuss the generalization of a slow–fast continuum of life histories across levels of biological organization, the various factors that can mask its expression within a population and the implications of our findings for the POLS hypothesis.

We relied on individual data to reconstruct the slow–fast continuum across species. By taking the average of individual life-history traits for each species, we assumed the data to be normally distributed, but this assumption was not always met (electronic supplementary material, S9; figures S7 and S8). We confirmed the robustness of our results by repeating the analysis using the median as well as the first and third quartiles of the data distribution (electronic supplementary material, figure S9). We nevertheless acknowledge that there may be uncertainties in the individual data due to, e.g. imperfect detection, variation in recapture rates and selective disappearance, and that the importance of such uncertainties is likely to differ among species.

A great challenge in transposing the slow–fast continuum from the across-species to the among-individual levels is that the overall variation in all life-history traits is much larger across species than among individuals. Indeed, the coefficient of variation in the four life-history traits we analysed was consistently larger across species compared to among individuals within populations, especially for adult lifespan, fecundity and age at first reproduction (electronic supplementary material, table S11). As hypothesized by Stearns in 1983 [4], as we descend along the taxonomic scale, the partitioning of the variation should change between taxa. The covariation between life-history traits would persist, but among fewer and fewer traits as some traits become fixed. Based on the set of species we analysed, our results also support this claim because we found an absence of individual variation in one or two life-history traits in three species (great tit, blue tit, and golden-mantled ground squirrel). Because the potential for variation in each life-history trait likely depends on the species, not all life-history traits may be equally relevant for the detection of individual patterns of variation across species.

Generalizing the concept of a slow–fast continuum of life histories across levels of biological organization (i.e. species, populations, and individuals) may be challenging, potentially because evolutionary and environmental constraints (e.g. body size, habitat quality) differ at each level and among species [15]. Our analyses revealed that the main axis of life-history variation among individuals within populations might differ depending on the species' position on the slow–fast continuum. The magnitude of individual variation within a population is likely not equal for all life-history traits and should differ between *slow* and *fast* species. For example, in *slow* species such as humans (with a generation time of *ca* 30 years), the potential to observe differences in lifespan between individuals is greater than in *fast* species such as white-throated dipper (with a generation time of less than 2 years). When focusing on the effect of individual heterogeneity alone, however, the opposite pattern is found: the variance is lower in lifespan for *slow* compared with *fast* species [38,39]. Therefore, finding a pattern of life-history variation that would fit all species is unlikely. General patterns may be easier to detect across species of similar generation times and their detection may require redefining, for a restricted range of generation times, the life-history traits that would capture the most individual variation.

There is still a possibility that a slow–fast continuum of life-histories exists among individuals within a population but is difficult to detect. Indeed, despite studies showing genetic correlations between life-history traits [40,41], a plethora of factors influence the phenotypic expression of genotypes in natural populations [15], which may prevent the detection of a slow–fast continuum. The slow–fast continuum consistently retrieved across species is generally interpreted as being brought about by species-specific differences in resource allocation to biological functions [42]. Evolutionary constraints in body plan, size, lifestyle and habitat requirements are much weaker among individuals within a population than across species. As a result, individuals within a population only differ slightly in energy allocation patterns but differ strongly in resource acquisition. Since van Noordwijk & de Jong's pioneering work [20], we know that higher variance in acquisition than allocation generates positive covariation among performance traits (see e.g. [13,43–45] in some of the species included in our analyses). These individual differences in resource acquisition explain why theoretical predictions seldom match empirical evidence in the POLS literature [29,46]. This widespread situation indicates that individual heterogeneity in resource acquisition confounds trait variation within a population. In addition, density dependence, which often occurs in vertebrate populations monitored for a long period, can modulate selective pressures on the speed of life at the population level [11,47], leading individuals to be faster at lower than higher population densities. Individual differences in resource acquisition and density-dependence are two processes illustrating the importance of considering the ecological and demographic context under which a population occurs when attempting to retrieve life-history trade-offs and the slow–fast continuum [27,48].

Two other important factors that might explain why there is no cross-level consistency, despite marked individual variation in the considered traits, are individual stochasticity and phenotypic plasticity. First, individual stochasticity corresponds to differences in life courses that are generated by the occurrence of random events in the life cycle of an individual (e.g. surviving or not, reproducing or not, etc.), and is thus the result of chance alone [49]. For instance, an individual allocating a lot of resources to reproduction may still live longer than an individual prioritizing allocation to maintenance because mortality may be due to random events whose occurrence is independent from the individual allocation strategies. Individual stochasticity is known to increase among-individual variation in life-history traits and to weaken correlation among traits [29]. Thus, stochasticity is likely an important source of variation in life-history traits generating unstructured individual variation in populations in the wild. Second, at the individual level, plasticity in the expression of genotypes may allow individuals to adjust their phenotypes according to environmental conditions. However, there is no reason to expect plastic responses to necessarily match evolutionary responses at the levels of populations or species and mismatches are likely to occur [24]. A careful examination of what shapes life-history variation at each level separately, from individuals to populations to species is thus needed.

Whether the lack of structure in individual life-history traits we found here corresponds to random individual variation or is the result of the coexistence of different processes or types of trait covariation cannot be teased apart in our analyses. Using a different analysis, Jenouvrier *et al*. [14] found that in addition to individual stochasticity, complex life-history patterns could be detected in the southern fulmar. Indeed, individuals were found to exhibit patterns of trait covariation in line with both the slow–fast continuum and a continuum of individual performance [50]. Specifically, a first group of individuals had late, but successful, recruitment and extended reproductive lifespan, a second group were less likely to recruit, had higher adult survival, and skipped breeding often, and a third group recruited early, attempted to breed often, and had a short lifespan. Individuals in the first and third groups performed better than individuals in the second group as they produced, on average, more offspring over their lifetime. The interplay between these two structuring axes of variation (slow–fast continuum and performance continuum; *sensu* Nussey *et al*. [51]) may interfere and prevent the detection of a unique main structuring axis of variation within populations.

The POLS hypothesis was first formulated to suggest covariations between physiological and life-history trade-offs among species and populations [15], and several studies have repeatedly supported its predictions (e.g. [16,52,53]). The POLS was later extended to incorporate expected covariations between behavioural and life-history traits, relying on the assumption that *slow* and *fast* phenotypes co-occur in a single population, opening a new branch of research on life-history variation within populations. However, this integrated POLS has so far received only limited empirical support [54], especially for vertebrates [55]. Indeed, out of 141 studies on vertebrates, support of the POLS was mixed [55]. This may be because the basic assumption of the POLS, i.e. the existence of a slow–fast continuum, does not hold. Considering that an individual slow–fast continuum should arise from a series of individual life-history trade-offs and that the latter have been proven difficult to detect in many taxonomic groups [48], this is not totally surprising. In our study, we go beyond single life-history trade-offs and specifically tested for the underlying assumption of the POLS, i.e. the existence of a slow–fast continuum. We showed little to no evidence of an individual slow–fast continuum within 17 populations of 17 different bird and mammal species. Our results show decisive evidence that the slow–fast continuum may not be the most appropriate framework on which to graft individual behavioural and physiological suites of traits, as the POLS hypothesis originally formulated [21]. It may remain possible to detect a generalizable slow–fast continuum among individuals in the wild in other taxonomic groups or once confounding factors such as demographic stochasticity, density dependence and individual resource acquisition will have been adequately accounted for. However, this task is far from easy because the relative importance of these confounding factors will vary across species and contexts [29].

In conclusion, our study revealed that the slow–fast continuum does not structure variation in individual life histories within bird and mammal populations as it does across species. Different species have different life cycles [1], but those differences may get blurred as we descend the taxonomic scale. For example, evidence of populations from the same species living in very contrasted environmental conditions showing both similar to as well as dissimilar life cycle speeds can be found [56,57]. Within populations, the variation among individuals appears much more diversified, involving multiple causes. Individual differences in resource acquisition and demographic stochasticity are likely to be dominant causes, but other biological processes may also come into play. In spite of this, individual heterogeneity in life histories is often detected and its impact on demography can be large (e.g. [14]). What governs individual differences in life-history traits is likely idiosyncratic across species, with the consequence that identifying a ‘one size fits all’ pattern of variation is unlikely. Quantifying and comparing the relative roles of chance and resource acquisition across species would help move forward in our understanding of what shapes individual variation in life histories within populations in the wild.

## Data Availability

Data are available from the Dryad Digital Repository: https://doi.org/10.5061/dryad.3bk3j9kpm [58]. Additional information is provided in the electronic supplementary material [59].
